# Continuous Renal Replacement Therapy in Critically-Ill Term and Preterm Newborns: A Single-Center Study in Belgrade

**DOI:** 10.3390/children12070828

**Published:** 2025-06-23

**Authors:** Snezana Rsovac, Katarina Milosevic, Brankica Spasojevic, Mirjana Cvetkovic, Gordana Milosevski Lomic, Biljana Medjo, Mina G. Cobeljic, Nadja Vukasinovic, Vesna Selakovic, Dusan Todorovic, Masa Petrovic, Davor Plavec, Jasna Kalanj

**Affiliations:** 1 Department of Pediatric and Neonatal Intensive Care, University Children’s Hospital, Tirsova 10, 11000 Belgrade, Serbia; 2 Faculty of Medicine, University of Belgrade, Dr. Subotića 8, 11000 Belgrade, Serbia; 3 Department of Allergology and Immunology, University Children’s Hospital, Tirsova 10, 11000 Belgrade, Serbia; 4 Department of Nephrology and Dialysis, University Children’s Hospital, Tirsova 10, 11000 Belgrade, Serbia; 5 Institute of Medical Physiology “Richard Burian”, Faculty of Medicine, University of Belgrade, Visegradska 26, 11000 Belgrade, Serbia; 6 Institute for Cardiovascular Diseases “Dedinje”, Heroja Milana Tepića 1, 11000 Belgrade, Serbia; 7 PRIMA NOVA, Healthcare Institution, 10090 Zagreb, Croatia; 8 Medical Faculty, JJ Strossmayer University of Osijek, 31000 Osijek, Croatia

**Keywords:** continuous renal replacement therapy, dialysis, neonates, prognostic factors

## Abstract

Background/Objectives: Continuous renal replacement therapy (CRRT) is an important treatment method that is becoming a commonly-used procedure in neonatal intensive care units (NICUs), especially in critically-ill neonates. Methods: We conducted a retrospective study aimed to evaluate factors influencing the outcomes of CRRT in neonates and preterm infants. The study analyzed data from 41 newborns treated with CRRT at our NICU over a ten-year period. Demographic, clinical, and laboratory parameters were assessed, including gestational age, birth weight, PRISM III score, and laboratory markers like urea, creatinine, and potassium levels, as well as characteristics of CRRT. Results: Our research found that the duration of CRRT, the presence of anuria, and higher potassium levels after initiation of CRRT were significant predictors of a poor outcome. Despite the lack of significant correlation between demographic characteristics, PRISM III score and the outcome, our findings highlight the importance of timely CRRT initiation and efficient management to improve survival. Conclusions: Our study identified several significant prognostic indicators in neonates undergoing renal replacement therapy. While these findings provide valuable insights, further research is needed to establish clear theoretical guidelines and improve clinical decision-making.

## 1. Introduction

Renal replacement therapy (RRT) has become a common procedure in neonatal intensive care units (NICUs), driven by the increasing perinatal survival rate and a growing number of critically-ill newborns requiring invasive therapeutic interventions [1,2]. Historically, peritoneal dialysis was the preferred modality of RRT due to its ease of use and ability to preserve vascular access. However, it was often inadequate in critically-ill neonates, particularly when rapid fluid removal was necessary [3,4]. Additionally, peritoneal dialysis is contraindicated in a significant number of conditions commonly observed in neonates [3].

In neonates, continuous renal replacement therapy (CRRT) is used as a life-saving intervention when conventional therapies fail to control acute kidney injury, severe fluid overload or electrolyte imbalance. In recent years, CRRT has emerged as the most widely used dialysis modality, as it can be effectively performed even in hemodynamically unstable newborns [4]. While CRRT is well studied in adults, data on its application in the neonatal population remain limited. Several studies have investigated factors influencing CRRT outcomes in neonates; however, a consensus has yet to be reached [5]. Therefore, the goal of our study was to identify prognostic factors of CRRT in newborns.

## 2. Materials and Methods

### 2.1. Participants

This retrospective cohort study included 41 neonates who underwent CRRT at the Neonatal Intensive Care Unit (NICU) of the University Children’s Hospital in Belgrade from 1 January 2014, to 31 December 2023. The study was approved by the ethics committee of the University Children’s Hospital in Belgrade (No. 017 16/70). The study was performed in accordance with the principles of the Declaration of Helsinki.

### 2.2. Inclusion and Exclusion Criteria

Participants were selected using retrospective purposive sampling from the electronic medical records. The inclusion criteria encompassed all neonates requiring CRRT due to acute kidney injury (AKI), volume overload, severe electrolyte imbalance, or drug intoxication during the study period. The exclusion criteria included neonates with severe congenital anomalies incompatible with life, withdrawal of care before CRRT initiation, and incomplete medical records. All neonates who met the inclusion criteria and had complete clinical documentation were included in the study.

AKI was defined according to modified KDIGO neonatal criteria, including criteria based on serum creatinine elevation and/or a urine output < 0.5 mL/kg/h for more than 6 h [6]. Anuria was defined as a urine output < 0.5 mL/kg/h for 24 h. The cut-off time for anuria before CRRT initiation was more than 24 h. Fluid overload was estimated using the fluid balance method and it was defined as %FO ≥ 10% over the baseline body weight. The primary indications for CRRT initiation were classified as anuria or volume overload, metabolic disturbance, i.e., intoxication or severe acidosis, and electrolyte imbalance. Both anuria and AKI were considered. The time to CRRT initiation, CRRT duration, and treatment modality (hemodiafiltration (HDF) vs. hemofiltration (HF)) were analyzed. CRRT-related complications including hemorrhage, hemodynamic instability, and dialysis circuit malfunction were recorded. Urine output before and after CRRT initiation was assessed as a marker of renal recovery.

### 2.3. Tools and Equipment Used

All neonates received CRRT using the Prisma Gambro device with the HF20 set. The priming of the circuit was performed with erythrocytes in 34 patients, human albumin solution in six patients, and fresh frozen plasma in one patient. The dialysis set was changed every 72 h or earlier if required. Both veno-venous and arterio-venous hemodialysis were utilized, with the most common vascular access sites being the femoral veins and arteries, jugular veins, and axillary arteries. In seven patients, peritoneal dialysis was performed either before or after CRRT blood priming was performed based on clinical assessment, particularly in patients with lower hematocrit values prior to CRRT initiation. Specifically, blood prime was used when hematocrit was below 40% or below the lower limit for age according to neonatal reference ranges, in order to avoid hemodilution and potential hemodynamic instability during CRRT initiation. All patients were maintained on heparin-based anticoagulation because in this period of study we did not use citrate anticoagulation CRRT.

### 2.4. Study Implementation

Demographic and clinical data were collected retrospectively from patient records, including sex, age at admission, gestational age and birth weight, body weight, primary disease, associated organ failure, prenatal and perinatal complications, and the need for inotropic support. The Pediatric Risk of Mortality III (PRISM III) score was calculated at admission to assess disease severity.

### 2.5. Laboratory Parameters

Laboratory parameters, including serum urea, creatinine, and potassium levels, were measured before and after CRRT initiation.

### 2.6. Statistical Analysis

Data analyses were conducted using SPSS 29.0 (IBM Corp., Armonk, NY, USA). Continuous variables were presented as mean ± standard deviation (SD) or median (25th–75th percentile), depending on their distribution, while categorical variables were expressed as counts and percentages. Comparisons between groups were performed using parametric tests (*t*-test) for normally distributed variables and nonparametric tests (Mann–Whitney U test and Chi-square test) for non-normally distributed variables. Pearson’s correlation coefficients were used to assess relationships between continuous variables. The Kaplan–Meier test was used to evaluate the difference in survival between groups. To evaluate the relationship between survival and independent variables, a Cox regression analysis was performed as univariate and multivariate analyses. *p*-values < 0.05 were considered statistically significant for all analyses.

## 3. Results

### 3.1. Patient Characteristics

During the ten-year observational period, a total of 41 neonates underwent CRRT at the NICU of our hospital (Table 1). All neonates were transferred from other institutions. The date of admission was defined as the first day of NICU stay at our center.

**Figure 1:**
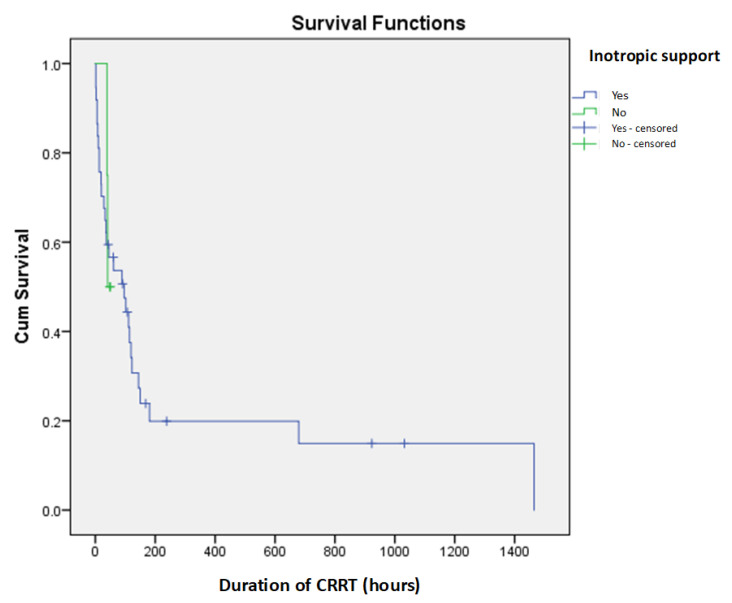
Kaplan–Meier survival curves for inotropic support as prediction of short-term mortality in newborns on CRRT.

The study cohort comprised 27 male (65.85%) and 14 female (34.15%) newborns. A fatal outcome occurred in 31 patients (75.6%), while 10 (24.4%) survived. The median gestational age of all patients was 35 weeks (IQR: 32–38), with 25 (60.97%) being preterm. The average birth weight was 2270 ± 780 g, with the lowest recorded birth weight at 760 g. The median age at admission was three days (IQR: 1–6). Surgical conditions were the most common cause of hospitalization, affecting twelve patients (29.27%), followed by kidney failure in eleven (26.83%), congenital heart disease in nine (21.95%), sepsis in four (9.76%), and hydrops fetalis and perinatal asphyxia in two patients each (4.88%), while one patient had an unspecified etiology. The median PRISM III score at admission was 13 (IQR: 9–18.5). All patients required mechanical ventilation at some point during hospitalization. The most frequently observed perinatal complication was asphyxia, reported in 12 patients (29.27%). Other perinatal complications included twin-to-twin transfusion syndrome (TTTSy), placental abruption, premature rupture of membranes (PROM), intrauterine growth restriction (IUGR), maternal COVID-19 infection, abnormal amniotic fluid levels, and maternal hypertension. Concomitant organ failure was documented in 27 neonates (65.85%), with respiratory insufficiency in 16 (39.02%), and heart failure in 15 (36.58%) being the most prevalent. No statistically significant differences were observed in demographic or clinical characteristics between survivors and non-survivors. However, gestational age showed a significant negative correlation with PRISM III scores (*p* = 0.019), indicating that younger neonates tended to have more severe illnesses upon admission. Although inotropic support was required in 37 patients (90.24%), it did not significantly impact survival outcomes (Figure 1).

**Figure 2:**
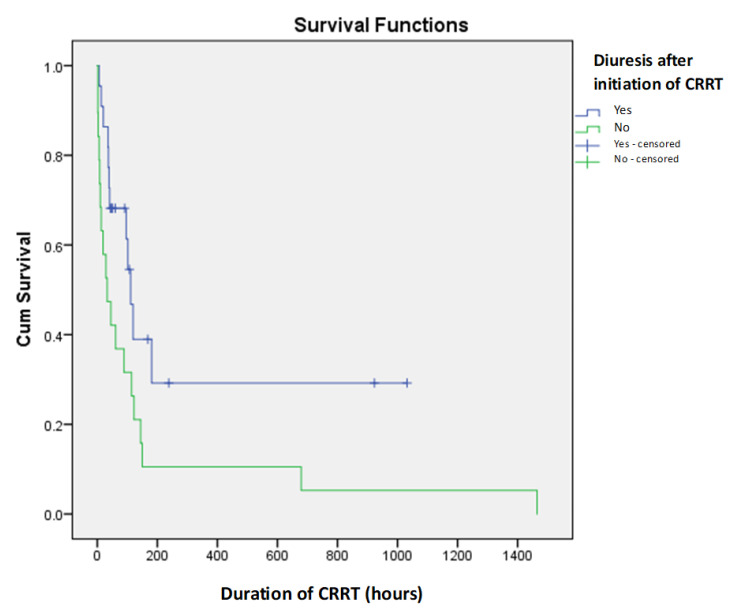
Kaplan–Meier survival curves for diuresis after initiation of CRRT as predictor of short-term mortality in newborns on CRRT.

There were no significant statistical differences between patients who received CRRT for less than 24 h and those who received CRRT for more than 24 h (*p* > 0.05), nor was there a significant statistical correlation between duration of CRRT and diuresis after initiation (*p* > 0.05) (Figure 2). This explains the observed negative correlation between CRRT duration and mortality (correlation coefficient r = −0.218).

### 3.2. Characteristics of Continuous Renal Replacement Therapy

CRRT was most commonly initiated due to anuria or volume overload, which was the primary indication in thirty-three patients (80.49%), followed by intoxication or severe acidosis in ten (24.39%) and electrolyte imbalance in six (14.63%) (Table 2). The median age at CRRT initiation was eight days (IQR: 4–13.5), with a mean duration of 159.88 ± 302.99 h (median: 50 h, IQR: 19.5–120.5). The median time from admission to CRRT initiation was 72 h (IQR: 18–138). A significant difference in CRRT duration was observed between survivors and non-survivors (*p* = 0.022), with a median duration of 99 h (IQR: 49.5–409.2) in survivors compared to 111 h (IQR: 61–150) in non-survivors. A CRRT duration of less than 24 h was identified as a predictor of a poor prognosis, with all 11 patients in this category experiencing fatal outcomes (*p* = 0.039).

**Figure 3:**
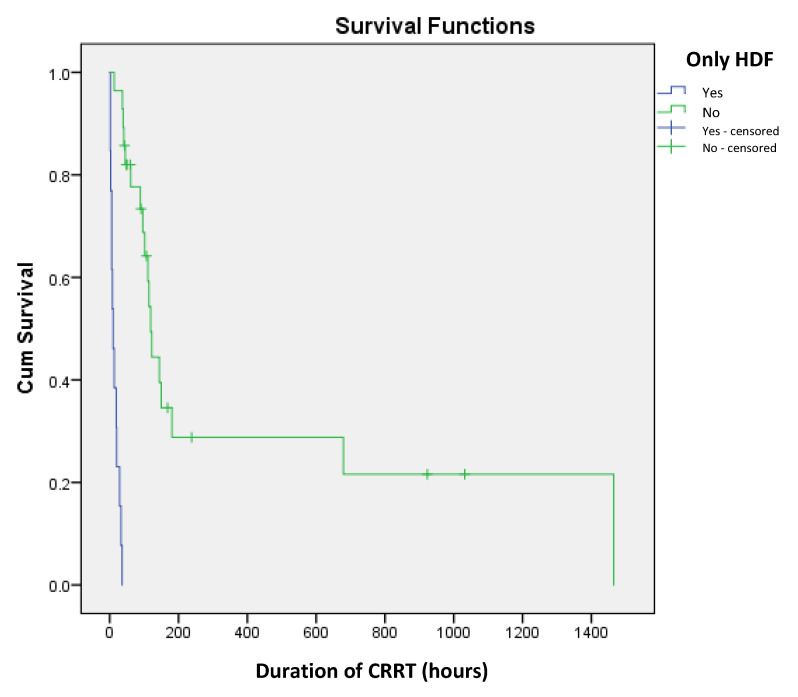
Kaplan–Meier survival curves for implementation of only HDF as predictor of short-term mortality in newborns on CRRT.

The choice of CRRT modality was also associated with significant differences in survival. Among the 13 patients who underwent HDF alone, mortality was 100% (*p* = 0.017). Conversely, in the 28 neonates who underwent both HDF and HF, the mortality rate was lower at 64.3%. Further analysis revealed that the duration of HF following HDF also had a significant impact on survival (*p* = 0.02). The median duration of HF in survivors was 44.5 h (IQR: 32.2–150.2), whereas in non-survivors, it was 70 h (IQR: 26–91) (Figure 3).

Anuria was noted prior to CRRT initiation in 25 patients. The absence of diuresis during CRRT was observed in 19 neonates, all of whom had a fatal outcome. Statistical analysis confirmed that persistent anuria during or after CRRT was a strong predictor of poor prognosis (*p* = 0.001). The presence of CRRT-related complications, including hemorrhage, hemodynamic instability, and dialysis set malfunction, was recorded in 33 patients. Among these, the mortality rate was 84.8%, significantly higher than the 37.5% mortality rate observed in the eight patients who experienced no complications (*p* = 0.013).

### 3.3. Laboratory Parameters

Laboratory parameters were assessed to determine their predictive value in clinical outcomes. Serum levels of urea and creatinine before and after CRRT initiation did not correlate with survival, nor did pre-CRRT potassium levels (Table 2). However, potassium levels after CRRT initiation were significantly higher in non-survivors compared to survivors (median: 3.5 mmol/L, IQR: 3.2–3.8 vs. median: 3.15 mmol/L, IQR: 2.9–3.72; *p* = 0.049). Furthermore, Cox regression analysis confirmed that post-CRRT potassium levels was an independent predictor of mortality (*p* = 0.004).

A multivariate analysis was conducted to assess the combined prognostic significance of CRRT duration, HF duration after HDF, and post-CRRT potassium levels. However, none of these parameters maintained statistical significance when analyzed collectively.

## 4. Discussion

Continuous renal replacement therapy in children is an emerging topic, as it represents the optimal method for acute renal replacement therapy and facilitates rapid volume reduction in critically-ill infants [7,8]. However, literature on CRRT in neonates and infants remains scarce, and survival rates are low, ranging from 15.15% to 66.7% [8,9,10,11,12,13]. It is important to note that most neonates requiring CRRT also have concomitant organ insufficiency beyond renal failure, as well as various perinatal complications [10,14,15]. The PRISM III score is commonly used to assess disease severity at admission [15], and it has been established that patients requiring CRRT often have high PRISM III scores [8]. Our findings confirm that the PRISM III score reflected the severe clinical condition of our patients; however, no correlation was observed between PRISM III scores and patient outcomes.

### 4.1. Predictors of Poor Outcomes

To identify potential predictors of poor outcomes, we analyzed demographic characteristics. Our study found that gestational age and body weight did not influence survival, a finding consistent with previous research [9,10,16]. However, while body weight and gestational age did not significantly impact outcomes in neonates, studies have shown that body weight is a key prognostic factor in the overall pediatric population [4,15].

Inotropic support was administered to 90.24% of patients in our study, yet it had no statistically significant impact on survival. The literature on this topic remains inconclusive, with studies presenting conflicting findings [4,9,15,16,17]. An interesting approach was presented in a study by Cortina et al., who calculated a vasoactive inotropic score at CRRT initiation, incorporating dosages of all vasoactive agents received by each patient. They found that this score was a significant prognostic factor for survival [18]. Future studies could further explore the prognostic value of vasoactive inotropic scores in neonates undergoing CRRT.

### 4.2. CRRT Duration and Timing

The optimal duration of CRRT in neonates remains uncertain, with reported averages ranging from 66 h to 15.6 days [9,10,11,12]. While previous research has largely focused on the technical feasibility and safety of CRRT, fewer studies have examined how treatment duration influences survival [19]. In our cohort, all neonates who received CRRT for less than 24 h had a fatal outcome, highlighting the importance of sustained therapy. Similarly, all patients who underwent only hemodiafiltration (HDF) as the initial CRRT mode had 100% mortality, suggesting that HDF alone may be insufficient in this population. We performed an additional statistical analysis to explore the correlation between duration of CRRT and patient outcome. Our results showed that in patients who died, CRRT was discontinued earlier due to death, which resulted in a shorter CRRT duration. This explains the observed negative correlation between CRRT duration and mortality (correlation coefficient r = −0.218).

A novel finding in our study was the impact of hemofiltration (HF) duration following HDF on survival outcomes. We observed that a longer duration of HF after HDF was associated with increased mortality, with survivors having a median HF duration of 44.5 h compared to 70 h in non-survivors (*p* = 0.02). This association has not been previously described in the literature. One possible explanation is that while HDF may correct electrolyte and metabolic imbalances early, persistent volume overload and inadequate urine output necessitate prolonged HF, which may reflect a more severe disease course. These findings highlight the need for close monitoring during the first 24 h of CRRT initiation, as this period appears to represent the highest risk window for mortality.

The timing of CRRT initiation remains a topic of debate. Our study found a median time from admission to CRRT initiation of 72 h (3 days), with only 26.8% of neonates diagnosed with kidney failure before NICU admission. There are no established guidelines for optimal CRRT initiation timing in pediatric patients [19]. However, recent studies indicate that delayed initiation is associated with increased mortality, with each hour of delay worsening outcomes [18,20,21]. Although we did not observe a statistically significant difference in survival based on CRRT initiation timing, we strongly advocate for the earlier recognition of AKI and fluid overload to facilitate timely intervention.

### 4.3. Laboratory Biomarkers in CRRT

Laboratory parameters were examined as potential prognostic markers. Serum urea and creatinine levels before and after CRRT initiation did not correlate with survival, consistent with a study by Noh et al. [19]. While serum creatinine is a widely used biomarker for AKI, it is influenced by maternal creatinine levels, gestational age, and renal immaturity, and its elevation often lags behind the onset of renal damage [3,14,22]. Consequently, there is a growing interest in identifying early biomarkers of neonatal AKI.

Emerging biomarkers such as cystatin C have been proposed as more reliable indicators of AKI in neonates, as they are less influenced by gestational age and maternal factors [23]. However, cystatin C was not routinely available in our hospital during the study period, representing a limitation of our analysis. The early detection of AKI using biomarkers could allow for earlier CRRT initiation, potentially improving outcomes.

Our study demonstrated that anuria and post-CRRT potassium levels are important predictors of poor outcomes. These findings highlight the clinical utility of monitoring diuresis and potassium levels as easily accessible bedside markers of CRRT efficacy and renal recovery [24].

CRRT in neonates and premature infants presents unique challenges, extending beyond clinical decision-making. Technical limitations remain a significant concern, particularly the lack of dialysis machines specifically designed for neonates. Many centers performing neonatal CRRT encounter difficulties in adapting standard dialysis devices for neonatal use [25]. Recognizing this issue, several European countries are actively working on developing and implementing CRRT devices specifically tailored for neonates and premature infants [26,27]. The availability of dedicated neonatal CRRT machines may significantly improve patient outcomes by offering greater precision in fluid management, ultrafiltration, and solute clearance.

### 4.4. Study Limitations

This study has several limitations. First, the relatively small sample size of 41 neonates and the single-center retrospective design may limit the generalizability of the findings and reduce the study’s statistical power to detect subtle but clinically relevant associations. Second, certain laboratory biomarkers such as serum cystatin C, which could provide more accurate assessments of renal function in neonates, were not available during the study period. Although all patients received CRRT using the same device and general protocol, variations in timing of referral and transport, selection of modality (HDF vs. HF), and supportive care strategies may have introduced treatment variability. Additionally, our analysis focused solely on in-hospital outcomes; long-term follow-up data regarding renal function, neurodevelopmental outcomes, and post-discharge mortality were not available. These limitations further support the need for prospective, multicenter studies with standardized protocols and extended follow-up.

## 5. Conclusions

Persistent anuria, CRRT duration, and dialysis modality may serve as important prognostic indicators in critically-ill neonates requiring renal replacement therapy. Despite the inherent limitations of our retrospective, single-center study, these findings offer meaningful insight into this underexplored field and provide a foundation for hypothesis generation in future prospective research. The study also contributes valuable information on CRRT-related complications—including hemorrhage, hemodynamic instability, and circuit malfunction—that are critical for clinical risk-benefit assessments. Furthermore, this work adds important regional data from Southeastern Europe, where experience with neonatal CRRT is still limited, thereby supporting broader international comparisons and informing future guideline development. The standardization of clinical protocols and the expansion of collaborative research networks will be essential for advancing neonatal renal support and improving outcomes.

## Figures and Tables

**Table 1:** Demographic and clinical characteristics of newborns.

		Total	Survivors	Non- Survivors	*p*-Value
Total (n, %)		41	10 (24.4%)	31 (75.6%)	
Gender (n, %)	Male	27 (65.85%)	5 (18.5%)	22 (81.5%)	0.267 ^a^
	Female	14 (34.15%)	5 (35.7%)	9 (64.3%)	
Gestational age in weeks (median, IQR)		35 (32–38)	36 (31.75–38)	35 (30–38)	0.818 ^b^
Prematurity (n, %)		25 (60.97%)	6 (24.0%)	19 (76.0%)	1.000 ^a^
Age at admission in days (median, IQR)		3 (1–6)	2.5 (1–7.75)	4 (2–6)	0.917 ^b^
Body weight at admission in kg (average, SD)		2.27 ± 0.78	2.48 ± 0.61	2.20 ± 0.84	0.360 ^c^
PRISM III score (median, IQR)		13 (9–18.5)	15 (5.25–18.25)	14 (12–23)	0.893 ^b^
Perinatal anamnesis (n, %)	Asphyxia	12 (29.27%)	2 (16.7%)	10 (83.3%)	0.694 ^a^
	TTTSy	5 (12.19%)	2 (40.0%)	3 (60.0%)	0.580 ^a^
	Placental abruption	4 (9.76%)	2 (50.0%)	2 (50.0%)	0.245 ^a^
	COVID- positive mother	1 (2.44%)	1 (100.0%)	0 (0.0%)	0.244 ^a^
	Oligohydramnios	4 (9.76%)	0 (0.0%)	4 (100.0%)	0.256 ^d^
	Polyhydramnios	3 (7.32%)	0 (0.0%)	3 (100.0%)	
	IUGR	2 (4.88%)	0 (0.0%)	2 (100.0%)	1.000 ^a^
	PROM	3 (7.32%)	1 (33.3%)	2 (66.7%)	1.000 ^a^
	Maternal hypertension	4 (9.76%)	0 (0.0%)	4 (100.0%)	0.556 ^a^
	No significant occurrences	11 (26.83%)	3 (27.3%)	8 (72.7%)	1.000 ^a^
Main cause of hospitalization (n, %)	Kidney failure	11 (26.83%)	3 (27.27%)	8 (72.72%)	
	Sepsis	4 (9.76%)	1 (25.0%)	3 (75.0%)	0.712 ^a^
	CHD	9 (21.95%)	1 (11.1%)	8 (88.9%)	0.410 ^a^
	Hydrops	2 (4.88%)	1 (50.0%)	1 (50.0%)	0.143 ^a^
	Asphyxia	2 (4.88%)	1 (50.0%)	1 (50.0%)	
	Surgical condition	12 (29.27%)	3 (25.0%)	9 (75.0%)	
	Other	1 (2.44%)	0 (0.0%)	1 (100.0%)	
Associated insufficiency of other organs (n, %)	Total	27 (65.85%)	4 (14.8%)	23 (85.2%)	0.064 ^a^
	Lungs	16 (39.02%)	2 (12.5%)	14 (87.5%)	0.265 ^a^
	Liver	8 (19.51%)	2 (25.0%)	6 (75.0%)	1.000 ^a^
	Heart	15 (36.58%)	2 (13.3%)	13 (86.7%)	0.277 ^a^
	GIT	3 (7.32%)	0 (0.0%)	3 (100.0%)	0.564 ^a^
Inotropic support (n, %)		37 (90.24%)	8 (21.6%)	29 (78.4%)	0.245 ^a^

IQR—interquartile range, SD—standard deviation, TTTSy—twin to twin transfusion syndrome, IUGR—intrauterine growth restriction, PROM—premature rupture of membranes, CHD—congenital heart disease, GIT—gastrointestinal tract, a—Fisher’s test, b—Mann–Whitney U test, c—T test for independent samples, d—Pearsons Chi-Square test.

**Table 2:** Characteristics of CRRT in newborns.

			Total	Survivors	Non- Survivors	*p*-Value
Indication for CRRT (n, %)	Anuria/volume overload		33 (80.49%)	7 (21.2%)	26 (78.8%)	0.378 ^a^
	Intoxication/acidosis		10 (24.39%)	2 (20.0%)	8 (80.0%)	1.000 ^a^
	Electrolyte imbalance		6 (14.63%)	2 (33.3%)	4 (66.7%)	0.622 ^a^
Anuria (n, %)	Before CRRT		25 (60.97%)	5 (20.0%)	20 (80.0%)	0.472 ^a^
	After CRRT initiation		19 (46.34%)	0 (0.0%)	19 (100.0%)	0.001 *^a^
Mode of dialysis (n, %)	Only HDF		13 (31.71%)	0 (0.0%)	13 (100.0%)	0.017 *^a^
	HDF and HF		28 (68.29%)	10 (35.7%)	18 (64.3%)	
Age at initiation of CRRT in days (median, IQR)			8 (4–13.5)	8 (3.5–22.75)	8 (5–10)	0.870 ^b^
Time from admission to initiation of CRRT in hours (median, IQR)			72 (18–138)	18 (1–294)	72 (48–120)	0.235 ^b^
Duration of CRRT in hours (median, IQR)			50 (19.5–120.5)	99 (49.5–409.25)	111 (61–150)	0.022 *^b^
Duration of HF after HDF in hours (median, IQR)			28.5 (0–73)	44.5 (32.25–150.25)	70 (26–91)	0.020 *^b^
Duration of CRRT (n, %)		> 24 h	30 (73.17%)	10 (33.3%)	20 (66.7%)	0.039 *^a^
		< 24 h	11 (26.83%)	0 (0.0%)	11 (100%)	
Complications during CRRT (n, %)		Yes	33 (80.49%)	5 (15.2%)	28 (84.8%)	0.013 *^a^
		No	8 (19.51%)	5 (62.5%)	3 (37.5%)	
Urea in serum (mmol/L) (average, SD)		Before HDF	11.2 ± 5.0	9.5 ± 5.40	11.7 ± 4.94	0.238 ^c^
		After HDF	4.7 ± 2.9	4.44 ± 3.05	4.8 ± 3.0	0.747 ^c^
Serum creatinine (umol/L) (average, SD)		Before HDF	170.7 ± 97.3	178.3 ± 141.17	168.2 ± 83.3	0.783 ^c^
		After HDF	79.4 ± 47.0	90.2 ± 62.7	75.4 ± 41.5	0.410 ^c^
K^+^ (mmol/L)		Before initiation of HDF (average, SD)	4.98 ± 1.27	4.85 ± 1.28	5.02 ± 1.31	0.718 ^c^
		After initiation of HDF (median, IQR)	3.50 (3.13–4.00)	3.15 (2.90–3.72)	3.50 (3.20–3.80)	0.049 *^c^

CRRT—continuous renal replacement therapy, HDF—hemodiafiltration, HF—hemofiltration, IQR—interquartile range, SD—standard deviation, K—potassium, *—statistical significance, a—Fisher’s test, b—Mann–Whitney U test, c—t-test for independent samples.

## Data Availability

The raw data supporting the conclusions of this article will be made available by the authors on request.

## Abbreviations

The following abbreviations are used in this manuscript:

CRRT	Continuous renal replacement therapy
NICU	Neonatal intensive care unit
RRT	Renal replacement therapy
AKI	Acute kidney injury
PRISM III	Pediatric Risk of Mortality III
HDF	Hemodiafiltration
HF	Hemofiltration
SD	Standard deviation
TTTSy	Twin-to-twin transfusion syndrome
IQR	Interquartile range
PROM	Premature rupture of membranes
IUGR	Intrauterine growth restriction
CHD	Congenital heart disease
GIT	Gastrointestinal tract
